# Unmet needs of Italian centers for pediatric diabetes care: analysis of a survey among pediatric diabetologists facing the national screening program for Type 1 Diabetes

**DOI:** 10.1186/s13052-025-01854-7

**Published:** 2025-03-13

**Authors:** Marco Marigliano, Roberto Franceschi, Enza Mozzillo, Valentina Tiberi, Monica Marino, Giada Boccolini, Malgorzata Wasniewska, Maria Elizabeth Street, Maria Rosaria Licenziati, Riccardo Bonfanti, Felice Citriniti, Giuseppe D’Annunzio, Maria Carolina Salerno, Valentino Cherubini, the Diabetes Study Group of the Italian Society of Pediatric Endocrinology and Diabetology (ISPED), Claudia Arnaldi, Giulia Berioli, Adriana Bobbio, Giulia Bracciolini, Francesca Cardella, Giuliana Marcella Cardinale, Vittoria Cauvin, Maria Susanna Coccioli, Anna Corò, Francesco Costantino, Fiorella De Berardinis, Valeria De Donno, Luisa De Sanctis, Elena Faleschini, Barbara Felappi, Francesca Franco, Alberto Gaiero, Francesco Gallo, Vanna Graziani, Lucia Paola Gueraggio, Dario Iafusco, Antonio Iannilli, Stefania Innaurato, Brunella Iovane, Alfonso La Loggia, Anna Lasagni, Nicola Lazzaro, Donatella Lo Presti, Fortunato Lombardo, Claudio Maffeis, Benedetta Mainetti, Chiara Mameli, Gianfranco Meloni, Sara Monti, Carlo Moretti, Filomena Pascarella, Elvira Piccinno, Gavina Piredda, Carmelo Pistone, Barbara Predieri, Ivana Rabbone, Emioli Randazzo, Petra Reinstadler, Carlo Ripoli, Irene Rutigliano, Andrea Scaramuzza, Riccardo Schiaffini, Laura Serra, Silvia Sordelli, Filomena Stamati, Letizia Tomaselli, Sonia Toni, Stefano Tumini, Maria Zampolli, Stefano Zucchini

**Affiliations:** 1 Department of Surgery, Dentistry, Pediatrics and Gynecology, Section of Pediatric Diabetes and Metabolism, University of Verona, Verona, Italy; 2 Department of Pediatrics, S.Chiara Hospital of Trento, APSS, Trentino-Alto Adige, Trento, Italy; 3 Department of Translational Medical Science, Section of Pediatrics, Università Degli Studi Di Napoli Federico II, Naples, Italy; 4 Department of Women’s and Children’s Health, Azienda Ospedaliero-Universitaria, Ospedali Riuniti Di Ancona, “G. Salesi Hospital”, Ancona, Italy; 5 Department of Human Pathology of the Adulthood and Childhood, University of Messina, Messina, Italy; 6 Department of Medicine and Surgery, University of Parma, Parma, Italy; 7 Neuro-Endocrine Diseases and Obesity Unit, Department of Neurosciences, Santobono-Pausilipon Children’s Hospital, Naples, Italy; 8 Unit of Pediatric Diabetology, Department of Pediatrics, Diabetes Research Institute, Vita-Salute San Raffaele University, Milan, Italy; 9 Azienda Ospedaliera Pugliese Ciaccio, Catanzaro, Italy; 10 Pediatric Clinic and Endocrinology, Regional Center for Pediatric Diabetes, IRCCS Istituto Giannina Gaslini, Genoa, Italy; 11 Azienda Ospedaliera Universitaria Integrata of Verona - Piazzale A. Stefani, Verona, 37126 Italy

**Keywords:** Type 1 diabetes, Care, Children, Adolescents, Benchmarking, Team, Screening, Technology

## Abstract

**Backgrounds:**

The incidence of Type 1 Diabetes (T1D) in children and adolescents is increasing by 3–4% per year. Children and adolescents with T1D (CwD) should receive person-centered, specialized treatment from a multidisciplinary team to ensure appropriate care. Italy is the first to implement a countrywide T1D screening program, which will raise the need for funding for specialized pediatric care. The study aims to update the organization of the Italian Centers for pediatric diabetes care.

**Methods:**

In 2022, members of the 59 Italian Centers following CwD were invited to complete an email survey regarding the Centers’ organization, characteristics, and activities. The questionnaire included information on responders, department organization, team composition, activities, and the organizational structures: department, ambulatory care services (AC), simple operational units (UOS), simple departmental operational units (UOSd), and complex operational units (UOC).

**Results:**

The data collected referred to the year 2022. According to the results, 21,318 people with diabetes were treated. Of these, 19,643 subjects (92.1%) have T1D (16,672 were CwD), 387 (1,8%) have Type 2 Diabetes, and 1,288 (6,1%) have other forms of diabetes. Compared to the 2012 survey, a 13% decrease (from 68 to 59 Centers) in the number of pediatric Centers caring for CwD was observed with a parallel increase of total (+ 6.6%) and average (+ 22%) number of CwD per Center. The estimated prevalence of T1D has increased (1.4 vs. 1.7 per 1,000 CwD—2012 vs. 2022). A reduction in numbers for AC (-22%) and UOS (-35%) was observed, whereas UOSd/UOC increased by 50%. Almost 35% of the dietitians and 40% of the psychologists were not permanent members of the multidisciplinary diabetes team.

**Conclusions:**

The observed decrease in the overall number of pediatric diabetes Centers, the reduction in specialized and dedicated HCPs, and the concurrent increase in the number of treated CwD in the last ten years indicate an alarming situation for pediatric diabetes treatment in Italy. Furthermore, the projected rise in CwD due to the National T1D screening program emphasizes the need for increased resources for specialized pediatric care of CwD at all stages.

**Supplementary Information:**

The online version contains supplementary material available at 10.1186/s13052-025-01854-7.

## Background

Type 1 Diabetes (T1D) is one of the most prevalent chronic metabolic diseases among children and adolescents. In recent decades, the global incidence rate has increased by 3 to 4% per year, with significant regional variations [1, 2]. In Italy, T1D is the most frequent type of diabetes in children and adolescents (93%), followed by monogenic diabetes (6%), while type 2 diabetes accounts for less than 1% of the cases [3]. The incidence of T1D in people aged 0–18 years has been reported at 13 per 100,000 person-years and prevalence at 1.4 per 1,000 [4]; more recently, a 33-year study found that the incidence of T1D in people aged 0–14 years increased from 12 per 100,000 in 1989 to 26.6 per 100,000 in 2021 [5].

Providing care for children and adolescents with T1D (CwD) can be challenging as it requires parents/caregivers and, in most cases, the entire family. To ensure appropriate care, CwD should receive specialized, person-centered care from a multidisciplinary team that includes diabetes-trained pediatricians, nurses with pediatric diabetes training, dietitians trained in pediatrics, and psychologists trained in pediatrics with expertise in childhood diabetes [6, 7]. Other professional figures, such as social workers and psychiatrists, are recommended as team members [8]. Moreover, structures of care should be assessed alongside processes and clinical and psychological outcomes [9]. At the same time, the continuous progress in the use of technology in diabetes management over the last decades [in particular, the use of continuous glucose monitoring (CGM), insulin pump (IP), and Automated Insulin Delivery systems (AID)] has radically changed the daily lives of CwD [10]. Technology has also enhanced the quality of life of CWD patients. Still, more specialized training is required to minimize one of the critical side effects of insulin therapy, such as hypoglycemia [11]. Continuous education and training for CwD and their parents/caregivers are critical for optimizing metabolic management, lowering cardiovascular risk factors, and preventing and/or slowing down chronic consequences. Micro and macro-vascular complications are the primary cause of morbidity, mortality, and resource utilization among people with T1D [12, 13].

Diabetes is primarily managed in outpatient or ambulatory settings, and structure indicators reflect how delivery systems are organized and funded [9, 14]. The size and structure of the diabetes care team’s local organization will be determined by available resources and geographical and demographic characteristics [15]. To ensure enough experience for the pediatric diabetes team, the Center should provide care to at least 150 CwD. To follow up with CwD living in remote areas, teams from district or regional Centers could organize outreach clinics [9].

The optimal number of diabetes care providers per 100 CwD has been previously suggested by the SWEET initiative (SWEET is an acronym derived from ‘Better control in Pediatric and Adolescent diabeteS: Working to crEate CEnTers of Reference) and the ISPAD (International Society for Pediatric and Adolescent Diabetes) guidelines to be 0.75–1.0 pediatric diabetologist, 1.0–1.25 diabetes nurse, 0.5 dietitians, 0.3 psychologists [6, 9]. In the last decades, several worldwide public and private health organizations and registries have focused on measuring and improving the quality of care for CwD [16–18]. Recently, the Italian Society of Pediatric Endocrinology and Diabetology (SIEDP) launched the ISPED-CARD initiative (Italian Society of Pediatric Endocrinology Diabetology Continuous clinicAl monitoRing of Diabetes) underling the importance of monitoring and continuous improvement of quality of care for CwD [19].

In 1987, Italy promulgated a law on diabetes prevention and care, which led to a reorganization of the Italian pediatric diabetes Centers. To improve care, in 2012, a “National Plan on Diabetes Disease” was approved; however, health organizations remain delegated to 20 individual regions, and the availability of local resources could be very different among them. Recently, the European Commission established the EDENT1FI program (https://www.edent1fi.eu/) to look at the potential of screening and follow-up in several countries. After receiving endorsements from major Pediatric Societies [20] and with the support of Fondazione Italiana Diabete, Italy has become the first country to pass a law enabling autoantibody screening for T1D and Celiac Disease (CD) for all children and adolescents [21]. The law established that pediatric primary care physicians are the first point of contact for families and primary screening, integrated with the pediatric T1D expert regional Centers where participants identified as at risk are referred to for follow-up and monitoring [22]. T1D screening and follow-up will require specialized pediatric care resources to assist children and adolescents in diabetes Centers [23], increasing the need for dedicated multidisciplinary teams.

The multidisciplinary diabetes team had only been implemented in a few Centers on the Italian territory, according to a 2012 survey [4]. Ten years later, this study produced an updated map of the organization and the availability of healthcare professionals (HCPs) in the Centers that follow up CwD.

## Methods

In June 2022, a questionnaire about the organization, characteristics of the HCP employed, and type of activities of the Centers was proposed by email to the 180 ISPED members working in 59 pediatric Centers. We omitted diabetologists of the adults in the survey, as recent data reported that in Italy, less than 3% of CwD under 15 years of age are followed out of pediatric Centers [24].

The Heads of the Pediatric Centers involved were invited to send back information regarding their Center organization and activities in diabetes care at the time of the deadline (July 2022). Fifty-nine (59) Heads of the Pediatric Centers answered the survey by November 2022; if the receivers did not return the questionnaire by the deadline, they were notified by email. The principal investigator (PI) obtained and processed incomplete questionnaires, contacting each Head of the Pediatric Centers individually via phone to assist them in completing the missing data. If questionnaires were coming from members of the same center (Head of the Center and other members), they were merged. When information was not homogeneous, PI directly contacted the Head to solve discrepancies. Consistency between the answers was evaluated, and it exceeded 90%.

### The questionnaire

The questionnaire is reported in the Supplementary material (S1) and includes personal information on responders (qualification, degree, specialties, position); department organization and team composition (multidisciplinary or not, full-time equivalents of the staff working in the diabetes center, type of contract, and who is financing); activities (number of children in care, breakdown by age and by type of diabetes, number of days dedicated to treating patients/week, staff availability to install diabetes technology, to deliver a 24 h telephone service and consultations for the pediatric ward).

### Organizational structures

Italy’s healthcare system is regionally based. The Italian National Health Service (INHS) provides universal coverage, essentially free of charge at the point of service. The INHS is organized into departments to optimize technical and human resources; each department requires coordination and comprises several units with comparable and/or complementary abilities that operate together. The department organization for the delivery of diabetes care to the pediatric population varies and differs from region to region and from hospital to hospital. For example, there are public hospitals with dedicated pediatric units for treating diabetes or diabetes teams that work in larger pediatric departments.

In the questionnaire, terms with the following meanings were used:

- *Center for pediatric diabetes*: a place where a child or adolescent (0–18 years) with T1D can be diagnosed and followed by pediatricians with experience in diabetes care, independently of the number of CwD followed in that center;
- *Department:* an organization that includes different units with similar and/or complementary skills working in an integrated way to optimize the use of technical and human resources; each department requires unique coordination;
- *Ambulatory care services [AC]* are clinics dedicated to specific healthcare activities;
- *Simple operational units [UOS]* represent functional structures of complex units and derive from specific articulations of clinical activities;
- *Simple departmental operational units [UOSd]* are structures created inside the department to organize and manage specific activities with responsibility and professional and organizational autonomy;
- *Complex operational units [UOC]* are organizational structures with all technical and professional activities that characterize a specific field. They have managerial autonomy, significant technical and instrumental equipment, and a relevant role in achieving the department’s aims.

UOC and UOSd were examined together because they represented a small sample. Moreover, the organization of the structures was distinguished as Hospital, University, or Outpatient according to the primary orientation of the single center.

### Statistical analysis

The Kruskal-Walis test was used to examine the distribution of HCPs by departmental organization. The distribution of centers according to patients was analyzed using the Fisher Exact test. Statistical analyses were performed using Jamovi (v. 2.5.2.0) software. The statistical significance was assessed using a level of probability lower than 0.05.

## Results

**Fig. 1:**
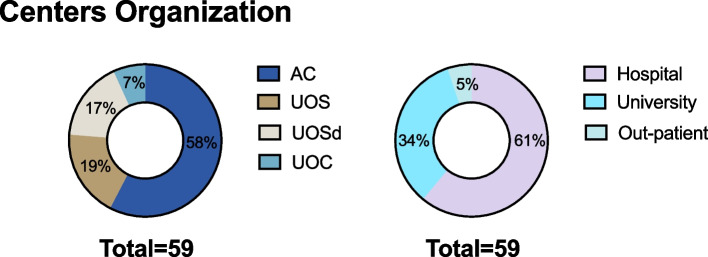
Classification of Italian Centers for the Treatment of Pediatric Diabetes in June 2022

**Fig. 2:**
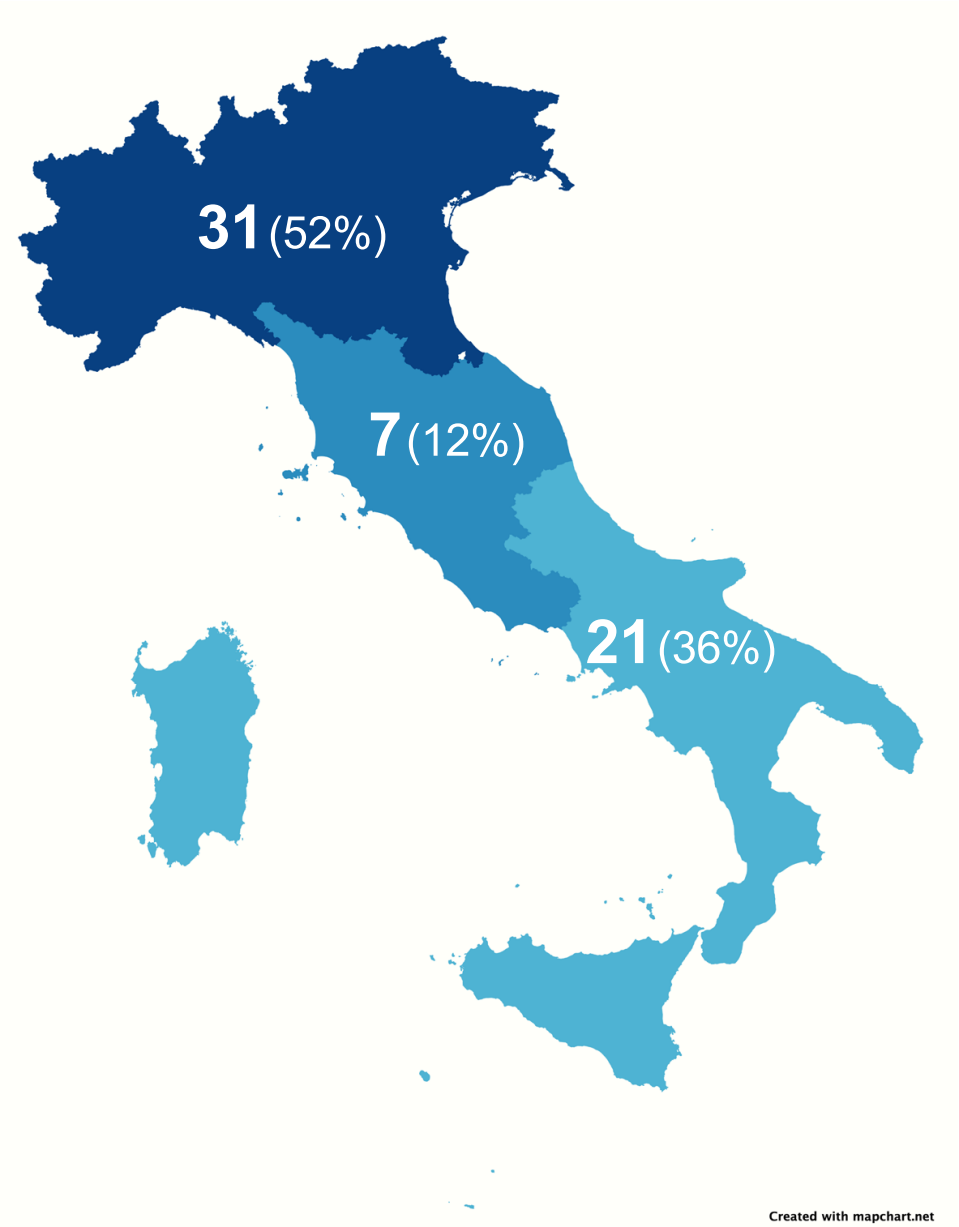
The numerosity of the Center (%) is determined by their geographical position (North, Center, and South + Islands)

Fifty-nine responses from 59 Centers (100%) were received, with data collected referring to 2022. The completeness of this ascertainment was 100% of all Centers following CwD. Overall, 34 Centers were classified as AC, 11 as UOS, 10 as UOSd, and four as UOC; 36 were Hospital, 20 were University, and 3 were Outpatient structures (Fig. 1). The center’s distribution according to the Region is shown in Table 1. Figure 2 shows the numerosity of Centers (expressed as absolute numbers and %) according to their geographical position (North, Center, and South + Islands).

**Table 1:** Distribution of pediatric Centers for diabetes in Italy in June 2022

Region	AC	UOS	UOSd	UOC	Total number of Centers	Total of children and adolescents (< 18 years) with T1D	Mean number per Center
**Valle d’Aosta**	1				1	32	32
**Piemonte**	3		1		4	1339	335
**Lombardia**	5	2	1		8	2176	272
**Liguria**	2				2	450	225
**Trentino-Alto Adige**	1		1		2	432	216
**Friuli Venezia Giulia**	1	1			2	227	113
**Veneto**	1	2		1	4	935	234
**Emilia Romagna**	8				8	1070	134
**Toscana**	1			1	2	1116	558
**Marche**				1	1	321	321
**Umbria**	1				1	188	188
**Lazio**		1	1	1	3	1600	534
**Abruzzo**			1		1	421	421
**Campania**	1	1	1		3	2465	822
**Puglia**	3	2			5	820	164
**Calabria**	3	1			4	306	76
**Sicilia**	1	1	3		5	1934	387
**Sardegna**	2		1		3	840	280
**Total**	34	11	10	4	59	16,672

### Number of patients

This survey shows that 21,318 people with diabetes were treated in the 59 Italian Centers. Of these, 19,643 subjects (92.1%) have Type 1 Diabetes, 387 (1,8%) have Type 2 Diabetes, and 1,288 (6,1%) have other forms of diabetes. In the population with Type 1 Diabetes, 16,672 subjects were 0–18 years old. The reported numbers of subjects were estimates provided by the Centers participating in the survey. The estimated prevalence of T1D was calculated to be about 1.7 per 1,000 people (population 0–18 years in 2022 in Italy: 9.788.622, http://dati.istat.it/). A breakdown by age and type of diabetes is presented in Table 2. About 85% of people with T1D followed in the Italian pediatric Centers are under 18, 13.6% are in the transitional age, and 1.5% are adults. The population of CwD enrolled was not normally distributed (Shapiro Wick test, *p* < 0.001); the median number of subjects < 18 years treated was 117 (IQR 117) in Centers classified as AC, 245 (IQR 281) in Centers classified as UOS, and 371 (IQR 363) in Centers classified as UOSd-UOC. The distribution of Centers according to the number of CwD < 18 years on follow-up is reported in Table 3, stratified by quartiles of the number of patients.

**Table 2:** Distribution by age groups of children and adolescents with Type 1 Diabetes and classification based on the form of diabetes

Class of Age	No = 19.643
** < 6 years**	2.052 (10.4%)
**6- < 12 years**	5.612 (28.6%)
**12–18 years**	9.008 (45.9%)
**18- < 25 years**	2.676 (13.6%)
** ≥ 25 years**	295 (1.5%)
**Diabetes type**	No = 21.318
**Type 1 diabetes**	19.643 (92.1%)
**Type 2 diabetes**	387 (1.8%)
**Other forms**	1288 (6.1%)

**Table 3:** Distribution of Centers stratified by quartiles of children and adolescents (< 18 years) with T1D. Fisher’s exact test

Number of centers (percentage)					
Number of CwD	Total	AC (*n* = 34)	UOS (*n* = 11)	UOSd/UOC (*n* = 14)	*p*-value
**Q1: ≤ 100**	17	13 (76%)	2 (12%)	2 (12%)	< 0.001
**Q2: 101–154**	13	11 (85%)	1 (7.5%)	1 (7.5%)	
**Q3: 155–397**	14	6 (43%)	4 (28.5%)	4 (28.5%)	
**Q4: ≥ 397**	15	4 (26.5%)	4 (26.5%)	7 (47%)	

### Centers organization

The 86% of the Centers have reported that pediatric patients with diabetes and other endocrine disorders were currently followed at the Center. More than 90% of the responding Centers provided inpatient and outpatient services. For Centers classed as AC, UOS, and UOC/UOSd, the average number of weekly days devoted to treating CwD was 3.25, 4.45, and 4.71, respectively. Most Centers (88%) provide a phone hotline with pediatric diabetologists on call 24/7; at smaller Centers, additional physicians who work in the same building may cover the service. More than 85% (51/59) of the Centers were directly involved in the education and training of CwD and their parents/caregivers, and the majority were very active in using technology to treat T1D.

### Healthcare Professionals (HCPs)

Table 4 displays the overall number and distribution of HCPs by department organization and the full-time equivalents (FTE) of working time dedicated to diabetes care. Of these HCPs, 8.3% of the physicians (11/132), 1.5% of the nurses (2/129), almost 35% of the dietitians (18/52), and 40% of the psychologists (16/40) were not a stable part of the multidisciplinary diabetes team and had annual contracts. Their contracts were primarily provided by hospitals (45% of physicians), regional government (33% of dietitians and 25% of psychologists), and no-profit family organizations (28% of dietitians and 56% of psychologists). Furthermore, only four Centers currently employ other staff members (secretary, educator, social worker, and biotechnician).

**Table 4:** Distribution of HCPs in absolute number and mean Full Time Equivalent (FTE)

		**Physician**	**Nurse**	**Dietician**	**Psychologist**
**Total**	**Absolute number Mean FTE**	132 / 0.56	129 / 0.49	52 / 0.25	40 / 0.17
**AC (*n*** **= 34)**	**Absolute number Mean FTE**	68 / 0.41	56 / 0.45	30 / 0.17	25 / 0.13
**UOS (*n*** **= 11)**	**Absolute number Mean FTE**	25 / 0.72	23 / 0.44	9 / 0.36	6 / 0.19
**UOSd/UOC (*n*** **= 14)**	**Absolute number Mean FTE**	39 / 0.81	50 / 0.67	13 / 0.38	9 / 0.21

**Fig. 3:**
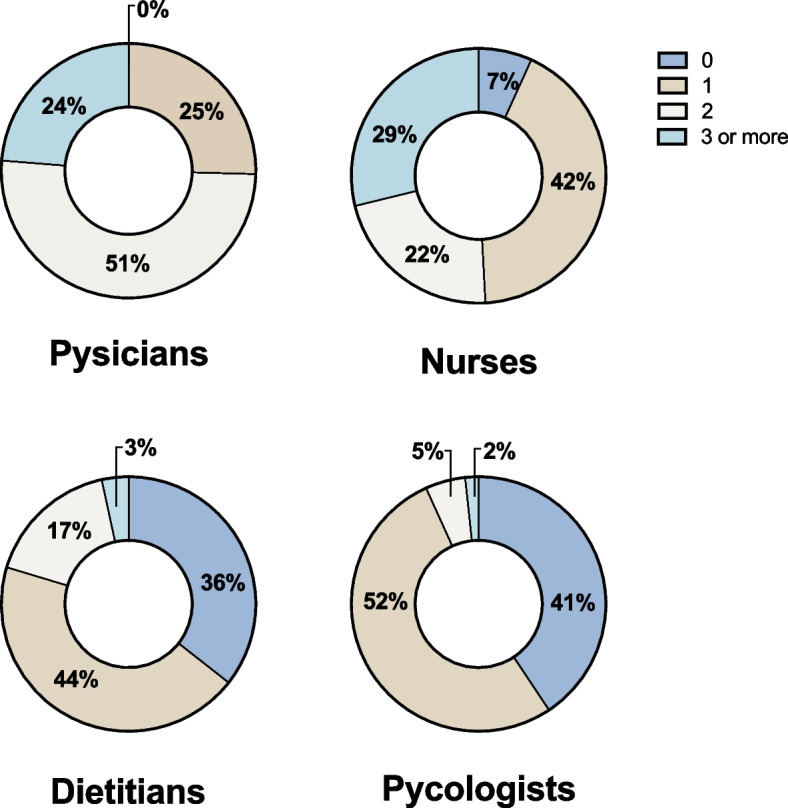
Percentages of Centers with 0, 1, 2, or ≥ 3 Healthcare Professionals (HCP: physicians, nurses, dietitians, and psychologists) working with children and adolescents with T1D

Figure 3 reported the percentages of Centers with 0, 1, 2, or ≥ 3 Healthcare Professionals (HCPs; physicians, nurses, dietitians, and psychologists) working with CwD. More than 1/3 of the Centers have no dietitians, and more than 40% have no psychologists. Table 5 reports the multidisciplinary team’s features for the number of CwD in follow-up, stratified by quartiles.

**Table 5:** Distribution of HCPs (number and FTE) stratified by quartiles of children and adolescents (< 18 years) with T1D treated

	**Staff composition in relation to the number of the patients in follow-up**				
Number of CwD	Physician (Mean number and FTE)	Nurse (Mean number and FTE)	Dietician (Mean number and FTE)	Psychologist (Mean number and FTE)	*p*-value
**Q1: < 100**	1.59 0.37	1.65 0.37	0.65 0.17	0.64 0.12	
**Q2: 100–154**	1.77 0.49	1.54 0.41	0.85 0.20	0.46 0.15	
**Q3: 154–397**	2.36 0.62	1.79 0.58	1.07 0.24	0.64 0.08	
**Q4: > 397**	3.27 0.81	3.73 0.59	1.00 0.38	0.93 0.30	

## Discussion

This survey was an update to the earlier one, which described the organization of pediatric diabetes care in Italy in 2012 [4]. Compared to the previous one, a 13% reduction in the total number of pediatric Centers taking care of CwD has been registered (from 68 Centers in 2012 to 59 Centers in 2022). Moreover, a concurrent increase of total (+ 6.6%) and average (+ 22%) CwD per Center was shown. These data should be considered a gross calculated estimate for several reasons: *i*. data were reported from the Centers participating in the survey; *ii*. the small number of CwD not under the care of the involved Centers was not included. An increase in the estimated prevalence of T1D was observed [1.4 vs 1.7 per 1,000 people (population 0–18 years 2012 vs 2022)]. As shown by Table 1 and Fig. 2, there was a significant variation in geographical distribution, organizational structure, and composition of the Centers. Despite having a larger geographical area, southern Italy had fewer pediatric diabetes treatment Centers than the north. Furthermore, no Centers have been identified in the Basilicata and Molise regions. These differences could be attributed to the region’s geographical features and regional healthcare organizations.

**Fig. 4:**
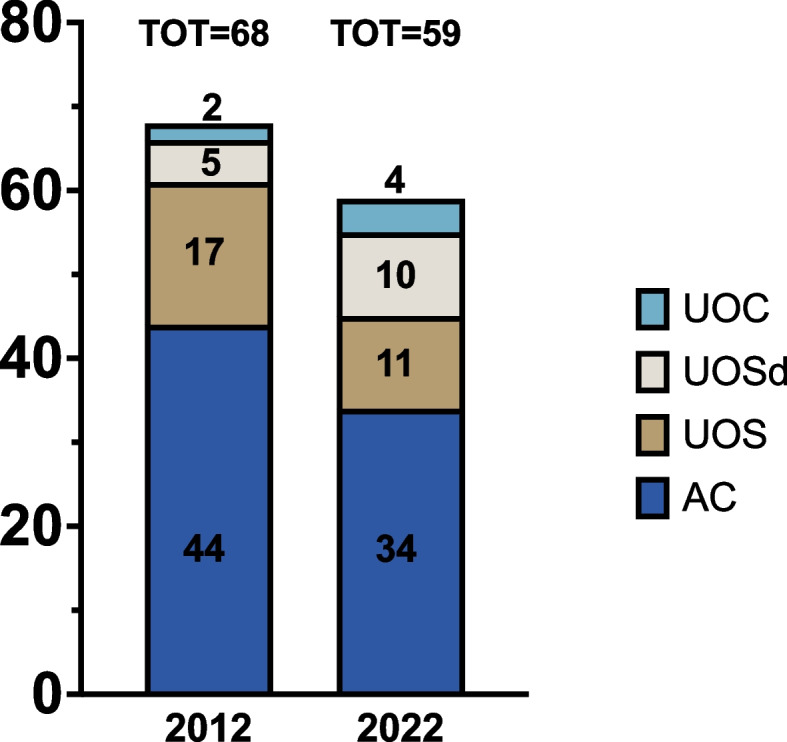
Distribution of the different pediatric Centers for treating pediatric diabetes in 2012 and 2022

**Fig. 5:**
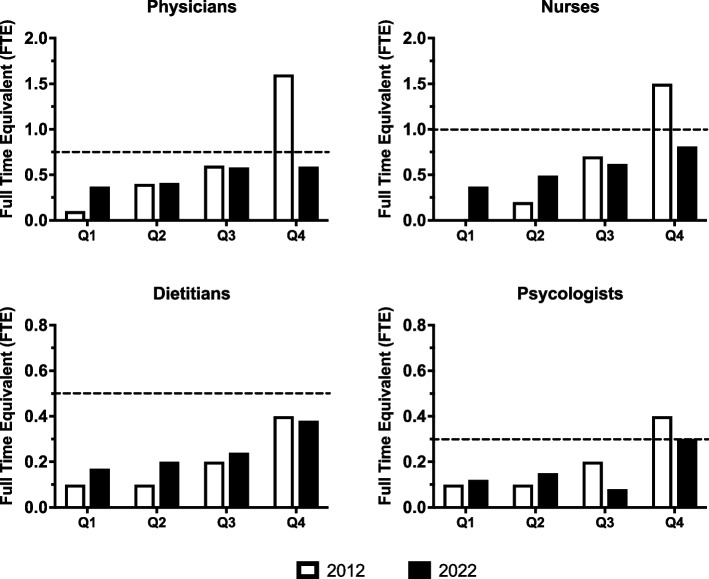
Distribution of mean HCPs Full Time Equivalent (FTE) stratified by quartiles of children and adolescents (< 18 years) with T1D treated in 2012 and 2022. Physicians (Panel **A**), Nurses (Panel **B**), Dietitians (Panel **C**), and Psychologists (Panel **D**). The dotted lines represent the minimum value of the recommended optimal FTE/100 CwD according to SWEET and ISPAD [6, 8]

Analyzing the numerosity and distribution of the four different organizational structures identified (AC, UOS, UOSd, and UOC), there was a reduction in numbers for AC (-22%) and UOS (-35%) with an increase of UOSd/UOC (+ 50%) (Fig. 4). Furthermore, the AC followed less CwD (38% following < 100, 32% following < 154), whereas the more organized Centers followed more CwD. A shortage of crucial professional figures for multidisciplinary care has been described, including pediatric diabetologists, specialized nurses, dietitians, and psychologists. In 2012, fewer than half of the Centers had a multidisciplinary team available, indicating major regional and structural variations in the care provided to CwD and their parents/caregivers. After ten years, the situation has not improved, and the FTEs of all the HCPs (physicians, nurses, dietitians, psychologists) in the multidisciplinary teams were way below the indicated threshold values [6, 8] (Fig. 5).

The role of dietitians and psychologists has grown in importance in educating CwD and their parents/caregivers, particularly with the huge increased use of technology (CGM, IP, and AID systems) for treating T1D [10]. The data analysis from this survey revealed a problematic condition, with more than one-third of the Italian Centers lacking dietitians and more than 40% lacking psychologists. Furthermore, many of these HCPs working in the multidisciplinary diabetes team (35% of dietitians and 40% of psychologists) were on annual contracts and not stable members. Moreover, despite the reduction in pediatric diabetes care Centers, the increasing number of new T1D diagnoses, and the demanding burden of technology for T1D treatment, very few CwD are treated in non-pediatric Centers [24].

We know from the literature that benchmarking analysis can confirm the high quality of care for CwD, particularly in pediatric Centers following national and international pediatric guidelines. The Italian Society of Pediatric Endocrinology and Diabetes (ISPED) through the ISPED-CARD initiative (Italian Society of Pediatric Endocrinology Diabetology Continuous clinicAl monitoRing of Diabetes) and the SWEET initiative through the registry [17] are thought to improve the quality of care in CwD, ensuring benchmarking analyses. Most of the responding Centers participate in national and international programs to measure and improve the quality of care for CwD.

Data from the international SWEET registry shows that switching from MDI to IP is significantly associated with improved glycemic control. Still, at the same time, it is necessary to pay more attention to nutritional education due to the risk of having a higher BMI-SDS [25, 26]. For these reasons, assistance from a multidisciplinary team of experts in pediatric T1D has been described as crucial, as it was associated with improved glycemic control and reduced complications [27]. Both the increasing use of technology in treating T1D and the presence of the multidisciplinary team underline the request for having more stable HCPs in the Italian pediatric diabetes Centers; trained and specialized HCPs should support the Centers to reach at least the minimum requested FTEs value considered necessary to maintain high standards of care.

Furthermore, with the adoption of the national screening program for T1D for the global pediatric population in 2023, the required resources for specialized pediatric care will increase. The national screening program has established that T1D expert regional Centers were responsible for education, autoantibody and metabolic monitoring, periodic visits, and overall assistance, including possible psychological assessment and support when needed for children and adolescents who underwent the screening [22]. According to the estimations, establishing a screening regimen combined with follow-up of children with early-stage T1D might eventually increase the overall number of children in pediatric treatment for T1D by 60% [23]. The additional care that has been suggested involves diabetes monitoring and counseling and, in some instances, anxiety treatment. These approximations have provided a basis for estimating the medical expenses linked to the extensive screening of children for T1D [28–30].

This survey has some limitations: *i.* the quality of care according to the average level of glycosylated hemoglobin, number of severe hypoglycemias, prevalence of complications, number of users of technologies in the Centers, etc., has not been evaluated; *ii*. data were not considering the patient’s and/or their caregiver’s opinions on the quality of care through dedicated questionnaires. However, the complete Italian situation of T1D care in pediatric age has been described with a 100% response rate from the Centers; moreover, comparing the results with data from the 2012 survey could identify the critical points that need improvement.

## Conclusions

The total number of pediatric diabetes Centers in Italy has decreased over the last decade. There has been a decline in the number of specialized HCPs, while the number of patients with CwD being treated has increased. This points to a significant trend in pediatric diabetes care across the country. Furthermore, the national T1D screening program is expected to increase the number of children at risk for diabetes, which highlights the urgent need for additional funding to offer specialized pediatric treatment for CwD at every stage.

## Supplementary Information

Supplementary Material 1.

## Data Availability

The datasets used and analyzed during the current study are available from the corresponding author upon reasonable request.

## Abbreviations

T1D: Type 1 Diabetes
UOC: Complex operational units
UOS: Simple operational units
UOSd: Simple departmental operational units
AC: Ambulatory care services

## References

[1] Gregory GA, Robinson TIG, Linklater SE, Wang F, Colagiuri S, de Beaufort C, Donaghue KC; International Diabetes Federation Diabetes Atlas Type 1 Diabetes in Adults Special Interest Group; Magliano DJ, Maniam J, Orchard TJ, Rai P, Ogle GD. Global incidence, prevalence, and mortality of type 1 diabetes in 2021 with projection to 2040: a modelling study. Lancet Diabetes Endocrinol. 2022 Oct;10(10):741–760. 10.1016/S2213-8587(22)00218-2. Epub 2022 Sep 13. Erratum in: Lancet Diabetes Endocrinol. 2022 Nov;10(11):e11. 10.1016/S2213-8587(22)00280-7. PMID: 36113507. 10.1016/S2213-8587(22)00218-2 36113507

[2] Tuomilehto J, Ogle GD, Lund-Blix NA, Stene LC. Update on Worldwide Trends in Occurrence of Childhood Type 1 Diabetes in 2020. Pediatr Endocrinol Rev. 2020 Mar;17(Suppl 1):198–209. 10.17458/per.vol17.2020.tol.epidemiologychildtype1diabetes. PMID: 32208564. 10.17458/per.vol17.2020.tol.epidemiologychildtype1diabetes 32208564

[3] Delvecchio M, Mozzillo E, Salzano G, Iafusco D, Frontino G, Patera PI, Rabbone I, Cherubini V, Grasso V, Tinto N, Giglio S, Contreas G, Di Paola R, Salina A, Cauvin V, Tumini S, d’Annunzio G, Iughetti L, Mantovani V, Maltoni G, Toni S, Marigliano M, Barbetti F; Diabetes Study Group of the Italian Society of Pediatric Endocrinology and Diabetes (ISPED). Monogenic Diabetes Accounts for 6.3% of Cases Referred to 15 Italian Pediatric Diabetes Centers During 2007 to 2012. J Clin Endocrinol Metab. 2017 Jun 1;102(6):1826–1834. 10.1210/jc.2016-2490. PMID: 28323911. 10.1210/jc.2016-2490 28323911

[4] Giorgetti C, Ferrito L, Zallocco F, Iannilli A, Cherubini V; Study Group for Diabetes of ISPED. Organization and regional distribution of centers for the management of children and adolescents with diabetes in Italy. Ital J Pediatr. 2015 Oct 8;41:74. 10.1186/s13052-015-0179-6. Erratum in: Ital J Pediatr. 2016 Mar 23;42:33. 10.1186/s13052-016-0245-8. PMID: 26449887; PMCID: PMC4598967. 10.1186/s13052-015-0179-6 PMC4598967 26449887

[5] Gesuita R, Rabbone I, Marconi V, De Sanctis L, Marino M, Tiberi V, Iannilli A, Tinti D, Favella L, Giorda C, Carle F, Cherubini V. Trends and cyclic variation in the incidence of childhood type 1 diabetes in two Italian regions over 33 years and during the COVID-19 pandemic. Diabetes Obes Metab. 2023;25(6):1698–703. 10.1111/dom.15024. (Epub 2023 Mar 9 PMID: 36810862). 36810862 10.1111/dom.15024

[6] de Beaufort C, Vazeou A, Sumnik Z, Cinek O, Hanas R, Danne T, Aschemeier B, Forsander G; SWEET group. Harmonize care to optimize outcome in children and adolescents with diabetes mellitus: treatment recommendations in Europe. Pediatr Diabetes. 2012 Sep;13 Suppl 16:15–9. 10.1111/j.1399-5448.2012.00908.x. PMID: 22931220. 10.1111/j.1399-5448.2012.00908.x 22931220

[7] Chiang JL, Maahs DM, Garvey KC, Hood KK, Laffel LM, Weinzimer SA, Wolfsdorf JI, Schatz D. Type 1 Diabetes in Children and Adolescents: A Position Statement by the American Diabetes Association. Diabetes Care. 2018 Sep;41(9):2026–2044. 10.2337/dci18-0023. Epub 2018 Aug 9. PMID: 30093549; PMCID: PMC6105320. 10.2337/dci18-0023 PMC6105320 30093549

[8] Chobot, Agata, Eckert, Alexander J., et al. Psychological Care for Children and Adolescents with Diabetes and Patient Outcomes: Results from the International Pediatric Registry SWEET, Pediatric Diabetes, 2023, 8578231, 9 pages, 2023. 10.1155/2023/8578231. 10.1155/2023/8578231 PMC12017242 40303253

[9] Limbert C, Tinti D, Malik F, Kosteria I, Messer L, Jalaludin MY, Benitez-Aguirre P, Biester S, Corathers S, von Sengbusch S, Marcovecchio ML. ISPAD Clinical Practice Consensus Guidelines 2022: The delivery of ambulatory diabetes care to children and adolescents with diabetes. Pediatr Diabetes. 2022;23(8):1243–69. 10.1111/pedi.13417. (PMID: 36537530). 36537530 10.1111/pedi.13417

[10] Cherubini V, Zucchini S, Bonfanti R, Rabbone I, Scaramuzza A. Which Treatment Modalities Are Being Used by Italian Children and Adolescents with Type 1 Diabetes? Diabetes Technol Ther. 2024;26(4):283–5. 10.1089/dia.2023.0510. (Epub 2024 Jan 22 PMID: 38252920). 38252920 10.1089/dia.2023.0510

[11] Zucchini S, Tumini S, Scaramuzza AE, Bonfanti R, Delvecchio M, Franceschi R, Iafusco D, Lenzi L, Mozzillo E, Passanisi S, Piona C, Rabbone I, Rapini N, Rigamonti A, Ripoli C, Salzano G, Savastio S, Schiaffini R, Zanfardino A, Cherubini V. Recommendations for recognizing, risk stratifying, treating, and managing children and adolescents with hypoglycemia. Front Endocrinol (Lausanne). 2024;4(15):1387537. 10.3389/fendo.2024.1387537. PMID:38894740;PMCID:PMC11183505. 10.3389/fendo.2024.1387537 PMC11183505 38894740

[12] Bjornstad P, Dart A, Donaghue KC, Dost A, Feldman EL, Tan GS, Wadwa RP, Zabeen B, Marcovecchio ML. ISPAD Clinical Practice Consensus Guidelines 2022: Microvascular and macrovascular complications in children and adolescents with diabetes. Pediatr Diabetes. 2022;23(8):1432–50. 10.1111/pedi.13444. (PMID: 36537531). 36537531 10.1111/pedi.13444

[13] Marigliano M, Lanzinger S, Zineb I, Barcala C, Shah AS, Svensson J, Tsochev K, Mazur A, Galli-Tsinopoulou A, Ioacara S, Jothydev K, Maffeis C. The role of sex on the prevalence of cardiovascular risk factors in children and adolescents with Type 1 diabetes: The SWEET international database. Diabetes Res Clin Pract. 2024;210: 111616. 10.1016/j.diabres.2024.111616. (Epub 2024 Mar 13 PMID: 38490494). 38490494 10.1016/j.diabres.2024.111616

[14] ElSayed NA, Aleppo G, Aroda VR, Bannuru RR, Brown FM, Bruemmer D, Collins BS, Hilliard ME, Isaacs D, Johnson EL, Kahan S, Khunti K, Leon J, Lyons SK, Perry ML, Prahalad P, Pratley RE, Seley JJ, Stanton RC, Gabbay RA, on behalf of the American Diabetes Association. 14. Children and Adolescents: Standards of Care in Diabetes-2023. Diabetes Care. 2023 Jan 1;46(Suppl 1):S230-S253. 10.2337/dc23-S014. PMID: 36507640; PMCID: PMC9810473. 10.2337/dc23-S014 PMC9810473 36507640

[15] Saiyed M, Hasnani D, Alonso GT, Richmond E, Besançon S, Cotterill A, Ngwu U, Mazza C, Rottembourg D, Lanzinger S; SWEET study group. Worldwide differences in childhood type 1 diabetes: The SWEET experience. Pediatr Diabetes. 2021 Mar;22(2):207–214. 10.1111/pedi.13137. Epub 2020 Oct 22. PMID: 33038056. 10.1111/pedi.13137 33038056

[16] Rossi MC, Nicolucci A, Arcangeli A, Cimino A, De Bigontina G, Giorda C, Meloncelli I, Pellegrini F, Valentini U, Vespasiani G; Associazione Medici Diabetologi Annals Study Group. Baseline quality-of-care data from a quality-improvement program implemented by a network of diabetes outpatient clinics. Diabetes Care. 2008 Nov;31(11):2166–8. 10.2337/dc08-0469. Epub 2008 Aug 11. PMID: 18694979; PMCID: PMC2571068. 10.2337/dc08-0469 PMC2571068 18694979

[17] Gerhardsson P, Schwandt A, Witsch M, Kordonouri O, Svensson J, Forsander G, Battelino T, Veeze H, Danne T. The SWEET Project 10-Year Benchmarking in 19 Countries Worldwide Is Associated with Improved HbA1c and Increased Use of Diabetes Technology in Youth with Type 1 Diabetes. Diabetes Technol Ther. 2021;23(7):491–9. 10.1089/dia.2020.0618. (Epub 2021 Feb 26 PMID: 33566729). 33566729 10.1089/dia.2020.0618

[18] Sandy JL, Tittel SR, Rompicherla S, Karges B, James S, Rioles N, Zimmerman AG, Fröhlich-Reiterer E, Maahs DM, Lanzinger S, Craig ME, Ebekozien O; Australasian Diabetes Data Network (ADDN); T1D Exchanged Quality Improvement Collaborative (T1DX-QI); Prospective Diabetes Follow-Up Registry Initiative (DPV). Demographic, Clinical, Management, and Outcome Characteristics of 8,004 Young Children With Type 1 Diabetes. Diabetes Care. 2024 Apr 1;47(4):660–667. 10.2337/dc23-1317. PMID: 38305782. 10.2337/dc23-1317 38305782

[19] Nicolucci A, Graziano G, Lombardo F, Rabbone I, Rossi MC, Vespasiani G, Zucchini S, Bonfanti R; ISPED CARD Study Group. Continuous improvement of quality of care in pediatric diabetes: the ISPED CARD clinical registry. Acta Diabetol. 2024 May;61(5):599–607. 10.1007/s00592-023-02233-6. Epub 2024 Feb 8. PMID: 38332378; PMCID: PMC11055792. 10.1007/s00592-023-02233-6 PMC11055792 38332378

[20] Cherubini V, Chiarelli F. Autoantibody test for type 1 diabetes in children: are there reasons to implement a screening program in the general population? A statement endorsed by the Italian Society for Paediatric Endocrinology and Diabetes (SIEDP-ISPED) and the Italian Society of Paediatrics (SIP). Ital J Pediatr. 2023;49(1):87. 10.1186/s13052-023-01438-3. PMID:37468976;PMCID:PMC10354886. 37468976 10.1186/s13052-023-01438-3 PMC10354886

[21] Bosi E, Catassi C. Screening type 1 diabetes and celiac disease by law. Lancet Diabetes Endocrinol. 2024;12(1):12–4. 10.1016/S2213-8587(23)00354-6. (Epub 2023 Dec 1 PMID: 38048797). 38048797 10.1016/S2213-8587(23)00354-6

[22] Cherubini V, Mozzillo E, Iafusco D, Bonfanti R, Ripoli C, Pricci F, Vincentini O, Agrimi U, Silano M, Ulivi F, D’Avino A, Lampasona V, Bosi E. Follow-up and monitoring programme in children identified in early-stage type 1 diabetes during screening in the general population of Italy. Diabetes Obes Metab. 2024;26(10):4197–202. 10.1111/dom.15779. (Epub 2024 Jul 26 PMID: 39054936). 39054936 10.1111/dom.15779

[23] Bonifacio E, Winkler C, Achenbach P, Ziegler AG. Effect of population-wide screening for presymptomatic early-stage type 1 diabetes on pediatric clinical care. Lancet Diabetes Endocrinol. 2024;12(6):376–8. 10.1016/S2213-8587(24)00101-3. (Epub 2024 May 6 PMID: 38723647). 38723647 10.1016/S2213-8587(24)00101-3

[24] Russo G, De Cosmo S, Di Bartolo P, Lucisano G, Manicardi V, Nicolucci A, Rocca A, Rossi MC, Di Cianni G, Candido R; AMD Annals Study Group. The quality of care in type 1 and type 2 diabetes - A 2023 update of the AMD Annals initiative. Diabetes Res Clin Pract. 2024 Jun 13;213:111743. 10.1016/j.diabres.2024.111743. Epub ahead of print. PMID: 38878867. 10.1016/j.diabres.2024.111743 38878867

[25] Cardona-Hernandez R, Schwandt A, Alkandari H, Bratke H, Chobot A, Coles N, Corathers S, Goksen D, Goss P, Imane Z, Nagl K, O’Riordan SMP, Jefferies C; SWEET Study Group. Glycemic Outcome Associated With Insulin Pump and Glucose Sensor Use in Children and Adolescents With Type 1 Diabetes. Data From the International Pediatric Registry SWEET. Diabetes Care. 2021 May;44(5):1176–1184. 10.2337/dc20-1674. Epub 2021 Mar 2. PMID: 33653821. 10.2337/dc20-1674 33653821

[26] Marigliano M, Eckert AJ, Guness PK, Herbst A, Smart CE, Witsch M, Maffeis C; SWEET Study Group. Association of the use of diabetes technology with HbA1c and BMI-SDS in an international cohort of children and adolescents with type 1 diabetes: The SWEET project experience. Pediatr Diabetes. 2021 Dec;22(8):1120–1128. 10.1111/pedi.13274. Epub 2021 Nov 9. PMID: 34716736. 10.1111/pedi.13274 34716736

[27] Sanal G, Shijin S, Krishna V, Kesavadev J, Basanth A, Krishnan G, Shankar A. Empowering Patients with Type 1 Diabetes through a Multidisciplinary Team-assisted, Technology-Enabled Education Program. Curr Diabetes Rev. 2023;19(4):e200522205073. 10.2174/1573399818666220520115420. (PMID: 35619301). 35619301 10.2174/1573399818666220520115420

[28] Besser REJ, Bell KJ, Couper JJ, Ziegler AG, Wherrett DK, Knip M, Speake C, Casteels K, Driscoll KA, Jacobsen L, Craig ME, Haller MJ. ISPAD Clinical Practice Consensus Guidelines 2022: Stages of type 1 diabetes in children and adolescents. Pediatr Diabetes. 2022;23(8):1175–87. 10.1111/pedi.13410. (Epub 2022 Sep 30 PMID: 36177823). 36177823 10.1111/pedi.13410

[29] Phillip M, Achenbach P, Addala A, et al. Consensus Guidance for Monitoring Individuals With Islet Autoantibody-Positive Pre-Stage 3 Type 1 Diabetes. Diabetes Care. 2024;47(8):1276–98. 10.2337/dci24-0042. Erratum.In:DiabetesCare.2024Nov1;47(11):2033. 10.2337/dc24-er11a. PMID:38912694;PMCID:PMC11381572. 38912694 10.2337/dci24-0042 PMC11381572

[30] Bell KJ, Brodie S, Couper JJ, Colman P, et al. Protocol for the Australian Type 1 Diabetes National Screening Pilot: Assessing the feasibility and acceptability of three general population screening models in children. Diabet Med. 2024 Nov;41(11):e15419. 10.1111/dme.15419. Epub 2024 Aug 11. PMID: 39129150. 10.1111/dme.15419 39129150

