# Supplementary material for: Unmet needs of Italian centers for pediatric diabetes care: analysis of a survey among pediatric diabetologists facing the national screening program for Type 1 Diabetes
Source: Ital J Pediatr. 2025 Mar 13;51:77. doi: 10.1186/s13052-025-01854-7 (PMC11907787; doi:10.1186/s13052-025-01854-7)
Supplement: Supplementary file 1 — Supplementary Material 1. [file 13052_2025_1854_MOESM1_ESM.pdf]

	DATA COMPILAZIONE
INFORMAZIONI SUL RESPONSABILE DEL CENTRO	COGNOME
	NOME
	TELEFONO CELLULARE
	EMAIL
	RUOLO
INFORMAZIONI SULLA STRUTTURA	PROVINCIA DI RESIDENZA DEL CENTRO
	COMUNE DI RESIDENZA DEL CENTRO
	NOME DELLA STRUTTURA
	TIPOLOGIA DELLA STRUTTURA
	COLLOCAMENTO
INFORMAZIONI SUL PERSONALE DIPENDENTE (IN RUOLO)	NUMERO TOTALE MEDICI OSPEDLIERI CHE OPERANO NEL CENTRO
	NUMERO TOTALE MEDICI UNIVERSITARI CHE OPERANO NEL CENTRO
	COGNOME E NOME MEDICO no. 1
	FTE MEDICO no. 1
	COGNOME E NOME MEDICO no. 2
	FTE MEDICO no. 2
	COGNOME E NOME MEDICO no. 3
	FTE MEDICO no. 3
	COGNOME E NOME MEDICO no. 4
	FTE MEDICO no. 4
	COGNOME E NOME MEDICO no. 5
	FTE MEDICO no. 5
	COGNOME E NOME MEDICO no. 6
	FTE MEDICO no. 6
	NUMERO TOTALE INFERMIERI CHE OPERANO NEL CENTRO
	FTE INFERMIERE no. 1
	FTE INFERMIERE no. 2
	FTE INFERMIERE no. 3
	FTE INFERMIERE no. 4
	FTE INFERMIERE no. 5
	FTE INFERMIERE no. 6
	NUMERO TOTALE DIETISTI CHE OPERANO NEL CENTRO
	FTE DIETISTA no. 1
	FTE DIETISTA no. 2
	FTE DIETISTA no. 3
	NUMERO TOTALE PSICOLOGI CHE OPERANO NEL CENTRO
	FTE PSICOLOGO no. 1
	FTE PSICOLOGO no. 2
	FTE PSICOLOGO no. 3
	NUMERO TOTALE ALTRI OPERATORI DI RUOLO CHE LAVORANO NEL CENTRO
	RUOLO ALTRO OPERATORE no. 1
	FTE ALTRO OPERATORE no. 1
	RUOLO ALTRO OPERATORE no. 2
	FTE ALTRO OPERATORE no. 2
	RUOLO ALTRO OPERATORE no. 3
	FTE ALTRO OPERATORE no. 3

PERSONALE A CONTRATTO	NOME E COGNOME MEDICO no. 1
	ENTE FINANZIATORE
	DURATA CONTRATTO (MESI)
	INFERMIERE no. 1
	ENTE FINANZIATORE
	DURATA CONTRATTO (MESI)
	DIETISTA no. 1
	ENTE FINANZIATORE
	DURATA CONTRATTO (MESI)
	PSICOLOGO no. 1
	ENTE FINANZIATORE
	DURATA CONTRATTO (MESI)
	ALTRO
	ENTE FINANZIATORE
	DURATA CONTRATTO (MESI)
PAZIENTI CON DIABETE IN CURA	NUMERO PZ IN CURA < 6 ANNI
	NUMERO PZ IN CURA 6 - <12 ANNI
	NUMERO PZ IN CURA 12 - < 18 ANNI
	NUMERO PZ IN CURA 18 - <25 ANNI
	NUMERO PZ IN CURA > = 25 ANNI
	NUMERO TOTALE PZ CON DT1
	NUMERO TOTALE PZ CON DT2
	NUMERO TOTALE PZ CON DIABETE GESTAZIONALE
	NUMERO TOTALE PZ CON ALTRE FORME DI DIABETE
ORGANIZZAZIONE PRESTAZIONI	QUANTI GIORNI ALLA SETTIMANA E' APERTO IL CENTRO
	IL CENTRO FORNISCE RECAPITO TELEFONICO AI PAZIENTI
DIABETE E TECNOLOGIA	CHI FORNISCE INFORMAZIONI SULLA TECNOLOGIA?
	CHI PROVVEDE ALL'IMPIANTO DELLE IP?
	CHI PROVVEDE ALL'IMPIANTO DEL CGM?
	CONTATTO PERSONALE AZIENDE CHE PRODUCONO IP CON PZ
	CONTATTO PERSONALE AZIENDE CHE PRODUCONO CGM
